# Predicting toxins found in toxin–antitoxin systems with a role in host-induced *Burkholderia pseudomallei* persistence

**DOI:** 10.1038/s41598-020-73887-3

**Published:** 2020-10-09

**Authors:** Brittany N. Ross, Joseph D. Thiriot, Shane M. Wilson, Alfredo G. Torres

**Affiliations:** 1 Department of Microbiology and Immunology, University of Texas Medical Branch, Galveston, TX 77555 USA; 2 Department of Pathology, University of Texas Medical Branch, Galveston, TX 77555 USA; 3 Present Address: Center for Microbial Dynamics and Infection, School of Biological Sciences, Georgia Institute of Technology, Atlanta, GA 30332 USA

**Keywords:** Microbiology, Bacteria, Pathogens

## Abstract

*Burkholderia pseudomallei* (*Bpm*) is a bacterial pathogen that causes Melioidosis, a disease with up to 40% mortality and an infection relapse of 15–23% despite antibiotic treatment. Ineffective clearance of *Bpm* by antibiotics is believed to be due to persistence, a hibernation-like survival mechanism modulated, in part, by toxin–antitoxin systems (TAS). Several organisms possess a repertoire of TASs but defining environmental cues eliciting their activity is hindered by laborious in vitro experiments, especially when there are many toxins with redundant function. Here, we identified which of 103 proteins in *Bpm* that share features found in toxins of the TAS and repurposed transcriptional data to identify which ones play a role in surviving intracellular host defenses. Putative toxins with the strongest transcriptional response were found to have low conservation between *Bpm* strains, while toxins that were constitutively expressed were highly conserved. Further examination of highly conserved toxins BPSS0899, BPSS1321, and BPSL1494 showed that they were functional, and their mutation led to reduce survival within macrophages and reduced in vivo persistence-associated pathology (abscesses) during treatment, but did not affect macrophages persistence. These findings highlight the utility of a data-driven approach to select putative toxins and suggests a selective role for some TAS in host survival.

## Introduction

*Burkholderia pseudomallei* (*Bpm*) is an opportunistic bacterial pathogen that can infect both animals and humans causing the disease Melioidosis. The estimated yearly predicted disease and mortality burden for *Bpm* infections is 169,000 cases and 89,000 (54%) deaths worldwide^1,2^. Without an existing vaccine, the only way to combat a *Bpm* infection is through a partially effective multi-antibiotic treatment that still results in high mortality rates and relapse in 15–23% of convalescent patients^2–4^. Although *Bpm* is intrinsically resistant to many antibiotics, emergence of resistance to clinically relevant antibiotics is rare, suggesting an alternative mode of bacterial survival^2,5–7^. Bacterial persistence has been associated with treatment failure of chronic infections caused by pathogens such as *Escherichia coli*, *Mycobacterium tuberculosis*, *Staphylococcus aureus*, *Acinetobacter baumannii*, *Pseudomonas aeruginosa,* and the fungal pathogen *Candida albicans*^8–11^. The mechanism of survival used in persistence differs from antibiotic resistance in that the bacteria are susceptible to antibiotics, but become transiently refractive to killing by reducing cellular functions and growth^12,13^. Previous publications have shown that *Bpm* has higher rates of persister cell formation than several other bacteria, reaching up to 10–64% when stationary growth phase bacteria are exposed to antibiotics^14–18^. Due to the high rate of *Bpm* persistence during infection, identification and targeting of persistence mechanisms offers a means to improve treatment efficacy.

Bacterial toxin-antitoxin systems (TAS) have been identified as one of the molecular switches that sense stress and in many cases activate a persistence phenotype. This phenomenon occurs using a variety of mechanisms to induce growth senescence. In most cases, TAS are composed of two cistronic genes, one encoding an antitoxin and another a toxin. During a favorable environment, the toxin is bound and neutralized by the antitoxin. Upon encountering a stressful stimulus, the toxin is freed from the antitoxin’s repression and becomes able to induce persistence by selectively inhibiting cellular processes^19,20^. Not all toxins are reversible, but those activated in persistence are, allowing for a dynamic population that responds to environmental stresses and recovers when the stress is removed.

Although the role of TAS in persistence induction has been controversial in non-pathogenic *E. coli*, there is strong evidence that these systems are playing a role in more complex organisms such as *Salmonella enterica* serovar Typhimurium, *M. tuberculosis*, and *Bpm*^14,21,22^. The TAS can respond to any number of environmental cues, including dehydration, nutrient limitation, bacteriophage infection, or nitric oxide release by macrophages^23,24^. The traditional approach to studying TAS is by over-expression of each toxin, often in a surrogate organism, and testing for induction of persistence via growth senescence in nutrient rich or minimal laboratory media^15,25^. As more toxins are being identified, this approach has become more laborious. Currently, there is no in silico predictive model to determine which environmental conditions might mediate toxin induction. To address this, we used a data mining approach and repurposed existing transcriptional data across 82 environmental conditions to determine which *Bpm* putative toxins are associated with survival in the host^26^. The overall goal was to generate a data-driven approach that directs selection of TAS for further investigation as mediators of persistence and their association with chronic infection and treatment failure.

## Results

### Identification of putative toxins

**Figure 1:**
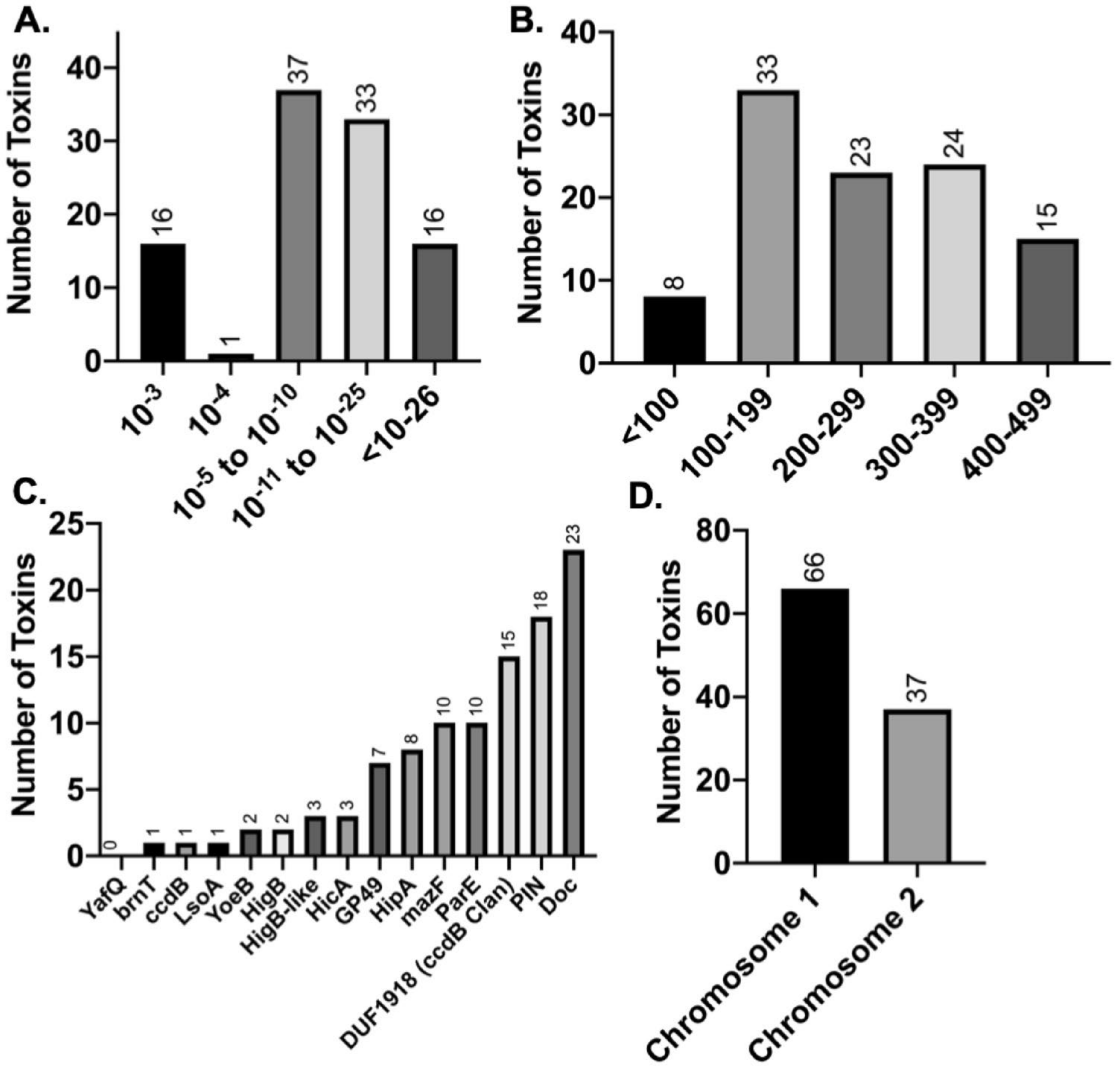
Putative toxins identified in *B. pseudomallei* K96243. Characteristics of the identified putative toxins are presented as: (**A**) expect value, (**B**) number of amino acids, (**C**) the toxin family they belong to, and (**D**) the chromosomal location.

To predict whether toxins have a role in host persistence, it is important to first determine how many putative toxins are encoded in the *Bpm* genome. Employing the same methodology used to find PIN domain-containing toxins, we PSI-Blasted all bacterial sequences contained in the Pfam for each of the 21 previously identified type II toxin families (Supplemental Table S1; Supplemental Figure S1). Using *Bpm* K96243 as the query, 103 predicted proteins containing toxin features were identified with an expect value of ≤ 0.001 (Fig. 1A) and we selected only those smaller than 500 amino acids^15,27–29^. The size cut off was derived from our knowledge that *Bpm* BPSS1584 is a functional toxin of 450 amino acids^15^. Two-thirds (60.2%) of the *Bpm* putative toxins were larger than standard toxin size of 200 amino acids (Fig. 1B)^27,28^ and the predominant classes identified were PIN (also known as VapC), and Doc toxins, representing 17% (n = 18) and 22% (n = 23) of all toxins, respectively (Fig. 1C). The toxins identified were asymmetrically distributed in the genome, with a heavier concentration in chromosome 1 (Fig. 1D), which generally encodes for core genes associated with central metabolism and growth, while chromosome 2 encodes accessory functions linked to adaptation and survival in different niches and virulence factors^30^. Although our goal was to streamline selection of toxins for experimental investigation, we also wanted to validate the functionality of the toxins identified. Using the traditional over-expression assay, we assessed functionality of eight toxins described in the next section and not previously investigated. Of the toxins BPSL0034, BPSL1494, BPSL2775, BPSL2851, BPSS0899, BPSS0698, BPSS0899, BPSS1321, and BPSS2196 that were tested, 75% of the over-expressed genes led to a reduction in growth, indicating their functionality (Supplemental Figure S2).

Next, we examined the overlap between our study and existing results from other experimental reports, computational programs, and databases. The most recent platform, TASmania, relies on 369 Hidden Markov Models (HMM; generalized protein sequence) to identify toxins^31^. TASmania identified 30 TAS with an expect value below 0.001, of which 8 (27.5%) were found in our current study (Supplemental Figure S3A and B). Re-running the RASTA computational program, we were unable to obtain the same results previously published^15^, and when compared the published results, we only had an overlap of 8 toxins (BPSL0549A, BPSL0562, BPSL1564, BPSL2333, BPSS0390, BPSS0845a, BPSS2142, and BPSS1226), which may be due to the stringent size and gene structure filters used by the RASTA analysis (Supplemental Figure S3A and B)^28^. Further, The overlap of data between RASTA and TASmania was also examined and showed low convergence. The Toxin-Antitoxin Database (TADB) is a compilation of all previously published computational and experimental results from which we found all toxins.

### Prediction of toxins important in the host using existing datasets

Historically, TAS have been investigated for their impact in antibiotic-mediated persistence but are also induced by environmental stress, such as low nutrients, high temperature, extracellular death factors, and immune system attacks^23,24^. Here, we predicted which toxins were host-induced by using an existing RNA expression dataset, exposing *Bpm* to 82 different conditions ranging from general laboratory growth, desiccation, survival in water, to in vivo infection (Supplemental Table S2)^26^. It is important to note that the dataset includes three wild type *Bpm* strains K96234, Bp22, and Bp008, and the latter two were included in the original publication as isogenic controls. *Bpm* K96243 and Bp22 share 94% genome with a 98% identity; however, the genome for Bp008 is not available.

**Figure 2:**
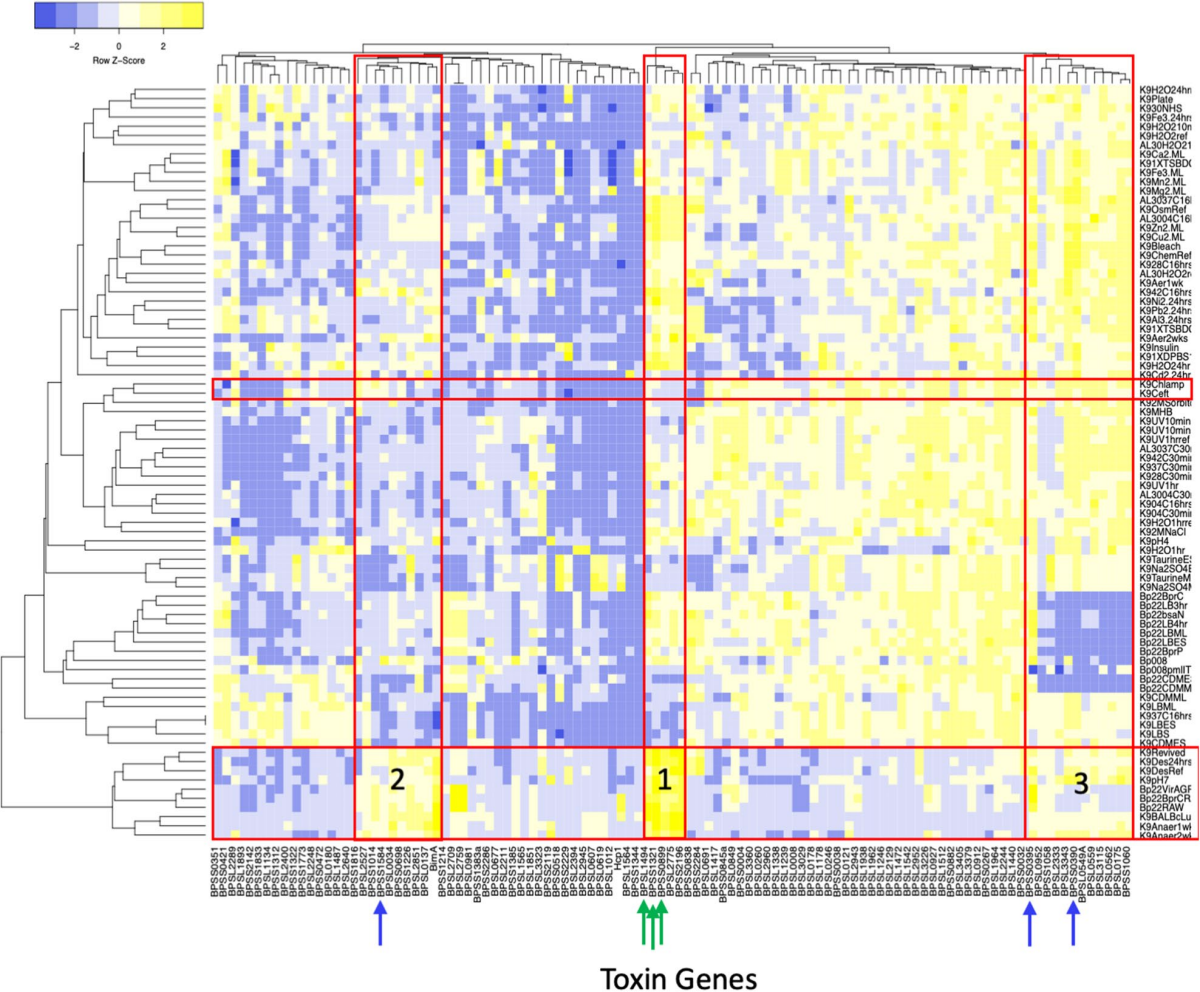
Predicting toxin-condition association. The dataset is presented as a heat map. Toxin genes are presented on the horizontal axis and conditions on the vertical axis. Both genes and conditions are clustered as shown on the top and left. Red boxes highlight antibiotic exposure (top) and exposure to host-like conditions (bottom), such as infection of RAW 264.7 macrophages, bacteria recovered from BALB/c mouse lung, pH 7, and exposure to anaerobic conditions. For ease, genes previously investigated are denoted with blue arrows and genes investigated in the current study are denoted in green arrows.

Hierarchical clustering identified seven sets of genes and seven clusters of conditions (Fig. 2). Of the putative toxins, 54% of the toxins are associated with most of the conditions tested and this finding could suggest redundancy in the toxin functions. Anaerobic conditions (strain K96243), in vivo lung infection (K96243), macrophage infection (Bp22) and pH 7 (K96243) are clustered together and represent conditions the bacteria would encounter in the host. Three gene clusters representing 26 genes were identified as being host-associated. Cluster one includes the novel putative toxins BPSS1321, BPSL1494, BPSS0899, BPSS2775, and BPSS2196. Seven of the genes in clusters 2 and 3 (BPS1584, BPSS0395, BPSL0559, BPSL2333, BPSS1060, BPSS0390, and BPSL0175) have been previously tested by either our lab or in the study by Butt et al*.* and some were shown to induce antibiotic-mediated persistence and influence persistence in a murine model of infection^14,15^. As controls, the type 6 secretion system (T6SS) subunit gene *hcp1* and the multi-nucleated giant cells-inducing gene *bimA* were included. Consistent with the literature, *bimA* and *hcp1* were induced during macrophage and in vivo infection^32–34^.

### Conservation and expression of the predicted host-associated toxins

We further evaluated toxins predicted to be important in the host-induced persistence phenotype and with a potential role in chronic infection. We hypothesize that for the gene product to play a role in a defined phenotype, it might be highly conserved within the bacterial species or isolates; therefore, we evaluated the conservation (presence/absence) of putative toxin genes in clusters 1–3 of *Bpm* and closely related *Burkholderia* strains (Table 1). Among 677 *Bpm* strains available in the database, putative toxins in clusters 1 and 2 were highly conserved. *B. mallei* (*Bm*) is the closest related species to *Bpm* and is believed to have originated following reductive evolution from *Bpm* to become a host restricted pathogen. We speculated that conserved toxins in *Bm* should be important for survival in the host. *B. mallei* displayed similar trends as *Bpm*, with higher toxin conservation in cluster 1 and the least conservation in cluster 3 (Table 1). Conversely, *B. thailandensis* shares 85% of the toxin genes with *Bpm,* and it is an environmentally restricted bacteria that has only caused a handful of reported cases of human illness^35^. As such, *B. thailandensis* had even fewer of the toxins conserved than *Bm*. To evaluate the conservation in a more distantly related species, we selected *B. cenocepacia*, which is notorious for causing chronic, life threatening infections in patients with lung abnormalities such as cystic fibrosis^36^. Interestingly, *B. cenocepacia* strains share only three of the putative toxins evaluated, BPSS0899, BPSL2527, and BPSS1584.

**Table 1:** Conservation of the Toxins from Cluster 1, 2, and 3.

	*B. pseudomallei (677)*	*B. mallei (65)*	*B. thailandensis (28)*	*B. cenocepacia (243)*
**Cluster 1**				
BPSL1494	100.00***	383.08***	100.00***	26.34**
BPSS1321	100.00***	100.00***	14.29*	0.00*
BPSS0899	100.59***	100.00***	185.71***	134.16***
BPSL2775	100.30***	1.54*	100.00***	1.65*
BPSS2196	366.03	327.69	450.00	302.06
**Cluster 2**				
BPSS1816	**100.00**	0.00	**100.00**	0.00
BPSL2527	**100.59**	**100.00**	**232.14**	**102.06**
BPSS1014	**100.44**	**100.00**	**100.00**	59.67**
BPSS1584	**99.41**	**290.77**	**189.29**	**115.23**
BPSL0034	**101.03**	44.62**	53.57**	7.41
BPSS0698	200.44	187.69	214.29	187.65
BPSS1226	**99.85**	**95.38**	0.00	0.41
BPSL2851	**101.33**	**98.46**	28.57**	4.12
BPSL0137	29.84**	0.00	3.57	0.00
BimA	617.28	332.31	328.57	134.16
**Cluster 3**				
BPSS0395	84.64***	0.00*	28.57**	0.00*
BPSL0952	13.59*	0.00*	7.14*	0.00*
BPSS1058	68.39***	0.00*	132.14***	30.04**
BPSL2333	99.41***	0.00*	60.71**	0.00*
BPSL3343	89.66***	0.00*	50.00**	7.41*
BPSS0390	5.76*	0.00*	35.71**	0.00*
BPSL0549A	10.04*	0.00*	0.00*	0.00*
BPSL0559	10.34*	0.00*	0.00*	0.00*
BPSL3115	18.91*	0.00*	0.00*	0.41*
BPSL0562	9.01*	0.00*	0.00*	9.88*
BPSL0175	48.74**	38.46**	32.14**	1.23*
BPSS1060	48.74**	38.46**	32.14**	1.23*

Nucleotide sequences for each putative toxin were BLASTed against all the available genomes for *Bpm*, *B. mallei, B. cenocepacia*, and *B. thailandensis* on the *Burkholderia* database. The cut-off value for homologues was an expect-value of 0.0001 and the number of hits was normalized on the total number of genomes for each species; Word size = 11 Filter = On Current DB Version: 8.1 (2018–04-30).

Bold indicates conserved in several *Burkholderia* species.

The percent conservation is depicted by asterisks based on high (***, > 75%), moderate (**, 25–75%), low conservation (*, < 25%), and broad homology (no asterisk).

In the processing steps of preparing the data for analysis and visualization, gene expression was min–max normalized to place all of them on the same scale. As with any data normalization, there are flaws and for min–max, it inflates the importance of constitutively active genes. Due to this, the standard deviation across all conditions was investigated to evaluate the overall gene expression (Table 2). Toxin expression was lowest in cluster 1 and highest in cluster 3. A closer look uncovered that cluster 1 was overly inflated with respect to their constitutive expression. When comparing expression to conservation, the data showed an inverse correlation (Table 2). Previously, we had investigated three of the toxin genes that had high expression and low-to-moderate conservation (BPSS0390, BPSS0395, and BPSL1584) and found moderate to no impact in host persistence^18^. Constitutively expressed toxin genes have not been investigated but owing to their high conservation, we chose to further evaluate for potential function of the putative toxins BPSS1321, BPSL1494, BPSS0899, BPSS2775, and BPSS2196, found in cluster 1.

**Table 2:** Association of conservation and standard deviation of expression.

Gene	Standard deviation of Log10 (raw reads)	Conservation
**Cluster 1**		
BPSL1494	0.24*	677 (100%)^^^
BPSS1321	0.25*	677 (100%)^^^
BPSS0899	0.26*	681 (100.5%)^^^
BPSL2775	0.27*	679 (100.2%)^^^
BPSS2196	0.22*	2478.00 ~
**Cluster 2**		
BPSS1816	0.76**	677 (100%)^^^
BPSL2527	0.38*	681 (100.5%)^^^
BPSS1014	0.28*	680 (100.4%)^^^
BPSS1584	0.49**	673 (99.4%)^^^
BPSL0034	0.21*	684 (101%)^^^
BPSS0698	0.25*	1357.00 ~
BPSS1226	0.24*	676 (99.8%)^^^
BPSL2851	0.30*	686 (101.3%)^^^
BPSL0137	0.50**	202 (29.8%)^
BimA	0.31*	4179.00 ~
**Cluster 3**		
BPSS0395	1.20***	573 (84.6%)^^
BPSL0952	1.45***	92 (13.6%)^
BPSS1058	0.77**	463 (68.4%)^^
BPSL2333	1.18***	673 (99.4%)^^
BPSL3343	1.64***	607 (89.7%)^^
BPSS0390	2.18***	39 (5.8%)^
BPSL0549A	1.19***	68 (10.0%)^
BPSL0559	1.16***	70 (10.3%)^
BPSL3115	1.69***	128 (18.9%)^
BPSL0562	1.45***	61 (9.0%)^
BPSL0175	1.66***	330 (48.7%)^^
BPSS1060	1.68***	330 (48.7%)^^

*** High SD, ** Moderate SD, * Low SD.

Conservation: Low (^), Moderate (^^), High (^^^), ~  Broad homology; Expect Value = 0.0001 Word size = 11 Filter = On Current DB Version: 8.1 (2018–04-30).

Toxins in clusters 1, 2 and 3 were analyzed for the conservation among other *Bpm* strains using Blast against 677 available genomes in the *Burkholderia* Database. Conservation is represented as a percentage of the total number of strains with the toxins. Genes that are homologous to broad families such as hydrolases or kinases are shown as ubiquitous.

### Over-expression of toxins in surrogate organisms

**Figure 3:**
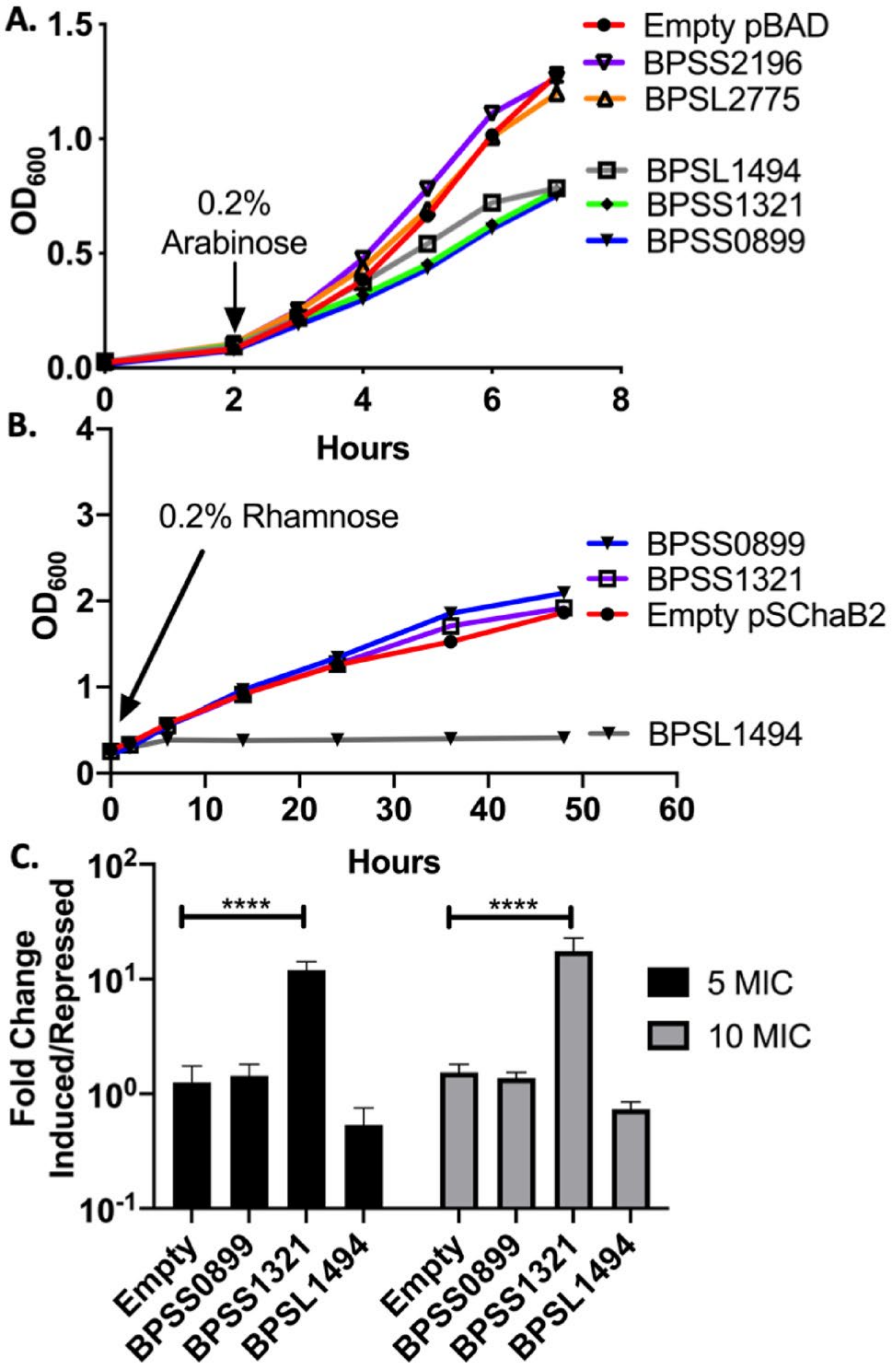
Overexpression of cluster one toxins. (**A**,**B**) Toxins were cloned into pBAD-myc-his or pSCRba2 plasmids and then transformed into either *E. coli* DH10b or *B. thailandensis*, respectively. After bacteria were grown in the corresponding media, cultures were divided into 6 tubes and toxin expression induced with (**A**) 0.2% arabinose (pBAD), (**B**) 0.2% rhamnose (pSCRba2), or mock treated with PBS and growth monitored by optical density. (**C**) To assay if toxin overexpression affected persistence, induced, and repressed *B. thailandensis* strains were treated with 5–10 × MIC of levofloxacin and represented as a fold-change between induced and repressed for each toxin. ****P < 0.0001.

Next, we over-expressed the genes in *E. coli* DH10B (Fig. 3A). When toxin expression was induced, BPSS0899, BPSS1321, and BPSL1494 reduced the growth rate of *E. coli* as compared to the control strain carrying the empty vector. Reduced growth indicated some form of growth arrest. We further tested whether BPSS0899, BPSS1321, and BPSL1494 could cause growth arrest in the closer related bacterial surrogate, *B. thailandensis*. When over-expressed, only BPSL1494 induced a reduction in growth compared to the strain carrying the empty plasmid (Fig. 3B). Interestingly, *B. thailandensis* carries orthologous toxin-antitoxin operons for BPSS1321 and BPSL1494, and over-expressed toxins’ activity may be abrogated by binding suppression of the orthologue antitoxin. As such, the bacteria were exposed to levofloxacin to promote toxin activation via release from antitoxin repression. After 24 h of antibiotic exposure, only expression of BPSS1321 conferred a survival advantage compared to the control strain (Fig. 3C). Although we found different results between *E. coli* and *B. thailandensis*, we chose to further investigate BPSS0899 (putative doc toxin), BPSS1321 (PemK/MazF-like), and BPSL1494 (doc toxin) in *Bpm* by allelic exchange mutagenesis (Supplemental Table S3).

### Non-host persistence phenotypes: swarming and antibiotic-induced persistence

Isogenic, unmarked mutants were constructed for the toxin genes BPSS0899, BPSS1321 and BPSL1494 to evaluate their function in the *Bpm* K96243 strain background. Typical assays to test persistence include swarming and antibiotic-induced persistence. Reduced swarming has been correlated to persistence in several pathogens^37–43^. All three toxin mutant strains had reduced swarming motility even when supplemented with a higher concentration of glucose (Supplemental Figure S4A). Antibiotic stress is typically the focus of persistence research; therefore, we carried out antibiotic-induced persistence assays and found reduced persistence of ΔBPSS1321 and ΔBPSL1494 in LB, but not RPMI (Supplemental Figure S4B and C). Conversely, ΔBPSS0899 resulted in increased persistence in RPMI, but not LB.

### Macrophage uptake, survival, and persistence

**Figure 4:**
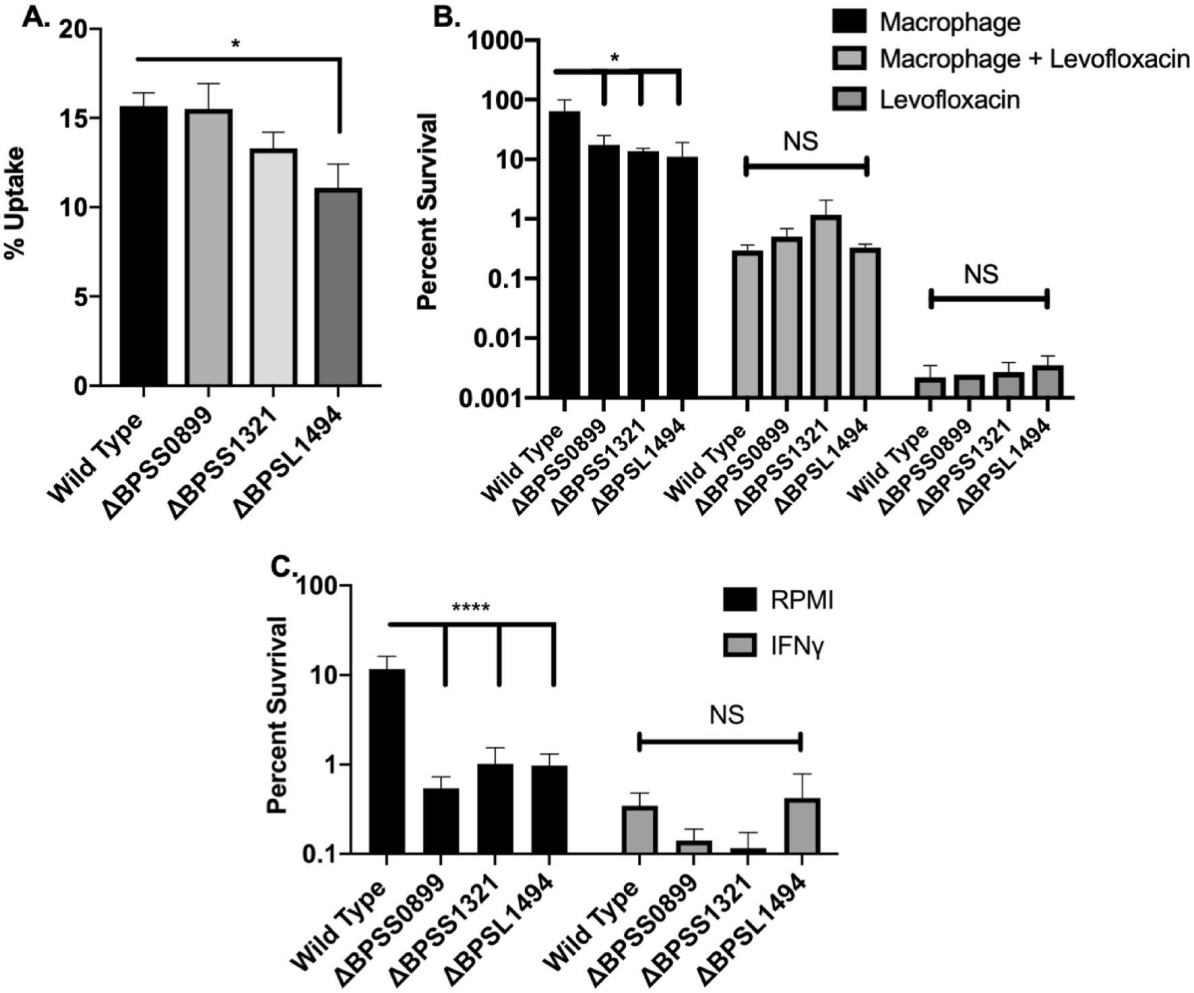
Invasion, survival, and persistence in macrophages. Macrophages were derived by incubating the U937 pleural monocytic cell line with PMA for 48 h. (**A**) Invasion, (**B**) survival, and persistence were assayed after 30 min of uptake followed by a 24 h incubation with or without 20 × MIC of levofloxacin. (**C**) To further investigate the role of macrophages on survival, U937 derived macrophages were polarized to a M1 phenotype using IFNγ for 16 h prior to infection and incubated for 24 h. One-way ANOVA was used to evaluate the difference within a treatment group. *P < 0.05, ****P < 0.0001.

Next, we evaluated the role of the toxins for persistence in host cells. When *Bpm* is in the host, macrophages are thought to be the preferred cell infected prior to establishment of a chronic infection^44^. Evidence shows that macrophages assist in dissemination as early as 24 h post infection and therefore, they are encountered early during infection^44^. U937-derived macrophages were infected and assayed for bacterial uptake, survival, and persistence. Significantly lower uptake was seen with only ΔBPSL1494 as compared to the wild type strain (Fig. 4A). All three mutants had lower ability to survive in macrophages, suggesting a defect in intracellular adaptation (Fig. 4B). However, when cell permeable antibiotic levofloxacin was added to kill actively growing intracellular bacteria (Fig. 4B), there was no significant difference in intracellular persistence between the mutants and the wild type strain. Compared to levofloxacin only treatment, macrophage exposure increased persistence as we have previously shown (Fig. 4B and^18^).

The reduced macrophage survival of ΔBPSS0899, ΔBPSS1321, and ΔBPSL1494 indicated a potential increase in susceptibility to innate antimicrobial killing found in these cells. Previous studies have shown that M1 polarized macrophages kill *Bpm* more efficiently than M0 differentiated macrophages due to NO production^45^. To evaluate the susceptibility of the mutants to M1 macrophage killing, U937 macrophages were differentiated with PMA then stimulated with IFNγ prior to infection. Activated cells were more readily able to kill *Bpm*; however, there was no significant difference between wild type and mutant strains (Fig. 4C).

### Simulating in vitro host-induced persistence

**Figure 5:**
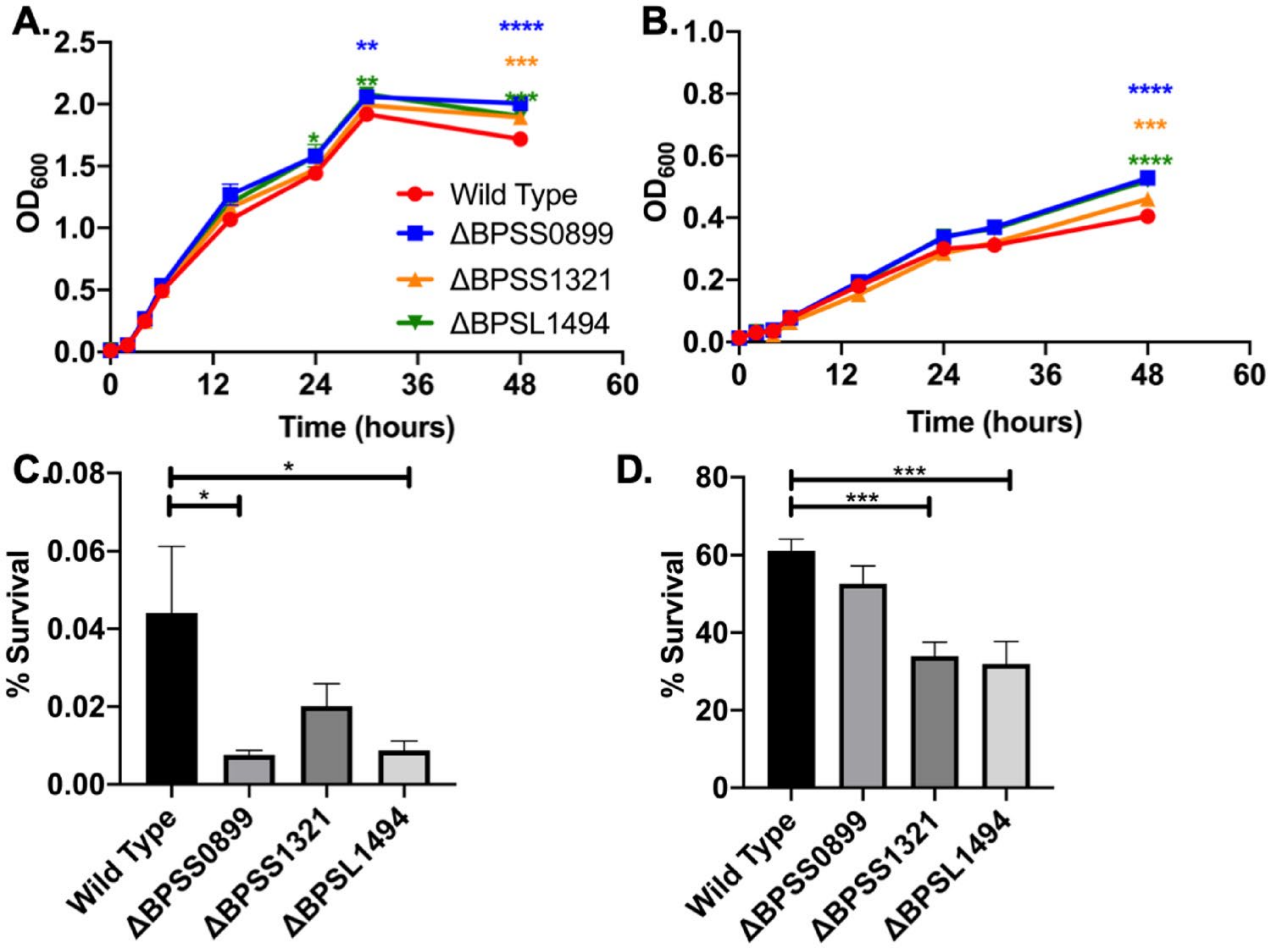
The effect of nutrient deprivation and nitric oxide stress on persistence. (**A**,**B**) The response to natural nutrient deprivation in late growth state was assayed by growing bacteria in either LB or RPMI and monitoring optical density and growth over 48 h. To test the impact of nutrient depravation further, bacteria were grown overnight in RPMI then exposed to (**C**) nutrient limitation for 30 min then treated with 20 × MIC of levofloxacin for 2 h. Survival was enumerated by normalizing to bacterial counts before antibiotics were added. In (**D**) a similar experiment was carried out with nitric oxide as the stress for 30 min prior to exposure to 20 × MIC of levofloxacin. One-way ANOVA was done with in each conditional group or between conditions for one strain. *P < 0.05, **P < 0.01, ***P < 0.001, ****P < 0.0001.

As we have shown, all three toxin mutants had a reduced capacity to survive in macrophages but behaved like wild type when levofloxacin was added in the macrophage-induced persistence assay. Two possibilities are feasible: (1) persistence is not affected and non-persister bacteria are being killed by the macrophage or (2) the toxins respond to host-stresses, but the levofloxacin treatment activates other TAS that compensate for the absence of the mutated toxins. To clarify this dichotomy, the effect of macrophage-like stress stimuli was assessed. Nutrient deprivation can induce persistence and occurs in some microenvironments within the macrophage which is a preferred niche for *Bpm*^18,44,46,47^. Nutrient deprivation also contributes to the reduction of bacterial growth in late growth stages. We hypothesize that if the toxins induce persistence during nutrient deprivation, loss of function could lead to higher bacterial titers at late growth stages. As hypothesized, all mutants tested grew to higher titers in both nutrient rich medium (LB) and a host-cell supportive, nutrient-defined, medium (RPMI) (Fig. 5A,B) suggesting a lack of nutrient deprivation recognition. We next tested survival in presence of antibiotics after nutrient starvation. Bacteria were grown in RPMI and then diluted and incubated in M9 minimal media supplemented with glycerol and casamino acids for 30 min prior to antibiotic exposure (Fig. 5C). Loss of BPSS0899 and BPSL1494 resulted in a significant defect in survival, while loss of BPSS1321 trended toward lower survival but was not statistically different than wild type.

During infection of macrophages *Bpm* is also exposed to nitric oxide (NO), which has been shown to increase *Bpm* persistence to the antibiotics imipenem and levofloxacin^18,45^. Exposure to NO for 30 min prior to levofloxacin treatment led to reduced survival when comparing ΔBPSS1321 and ΔBPSL1494 to wildtype (Fig. 5D). It is important to note that with antibiotics alone, ΔBPSS0899 survived better than wild type (Supplemental Figure S4C), but it did not when pre-treated with NO (Fig. 5D).

### In vivo persistence

**Figure 6:**
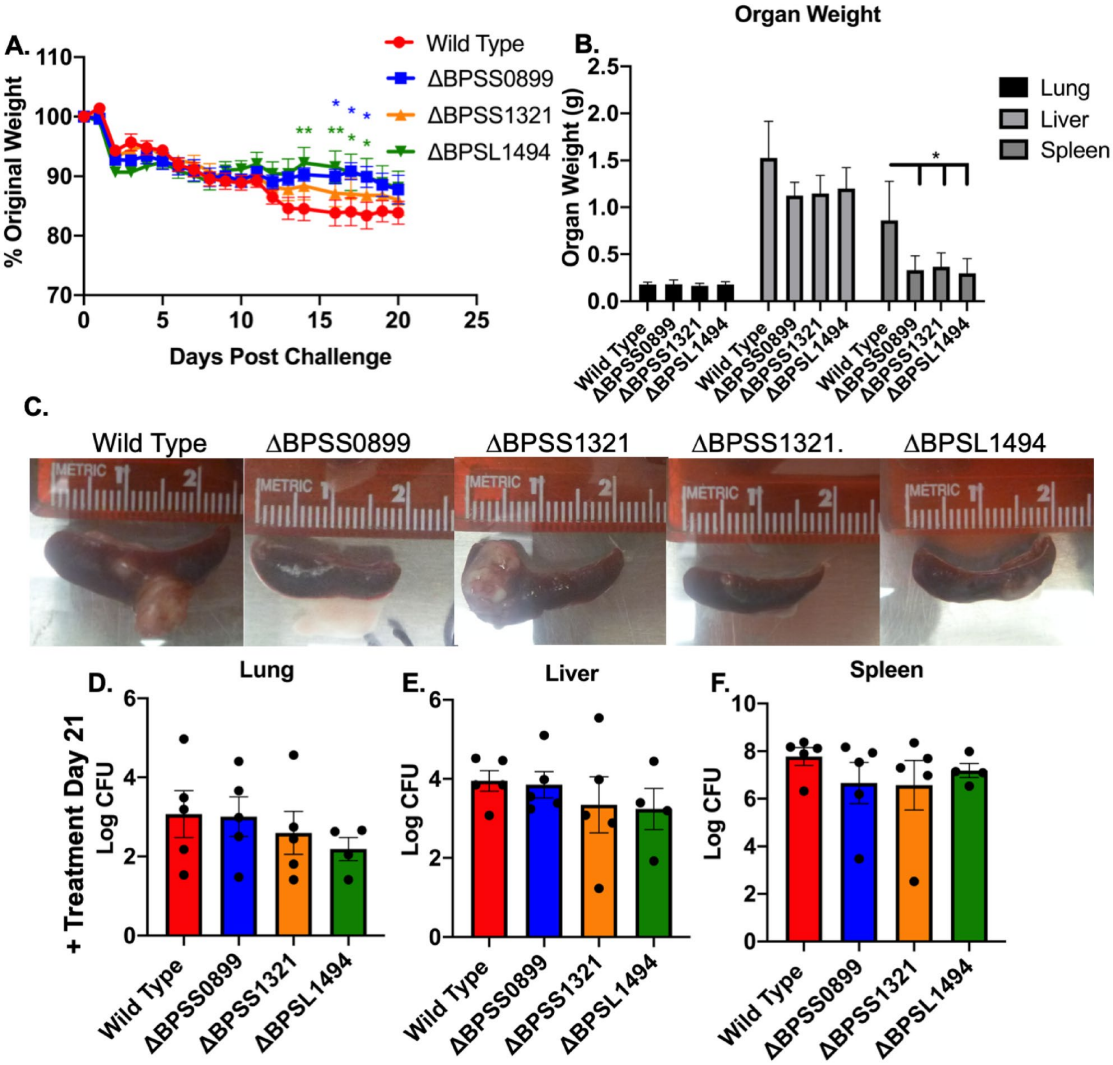
In vivo Persistence of ΔBPSS0899, ΔBPSS1321, and ΔBPSL1494 Following Treatment. Mice were intranasally infected with 3.5 CFU equivalent of each mutant and treated for 5 days with 25 mg/kg/day of levofloxacin. (**A**) Weight was monitored for the 20-day infection period after which organs of surviving mice were collected, (**B**) weighed, and (**C**) visualized for gross pathology. (**C**) Representative images are displayed, and two images were selected from ΔBPSS1321 due to variability in pathology. (**D**) Lungs, (**E**) livers, and (**F**) spleens were homogenized and bacteria cultured on agar to assess bacterial burden. Statistics were completed using Two-Way ANOVA or One-Way ANOVA. *P < 0.05 and **P < 0.01.

In our prior publication, we showed that toxin mutants ΔBPSS0390 and ΔBPSS0395, found in clusters 2 and 3, showed reduced bacterial colonization upon infection of BALB/c mice and treatment with levofloxacin^18^. Testing colonization of ΔBPSS0899, ΔBPSS1321, and ΔBPSL1494 after infection and levofloxacin treatment resulted in transiently (days 14–18) higher weights of mice infected with ΔBPSS0899 and ΔBPSL1494 as compared to wild type (Fig. 6A). At the end of the experiment, the spleens of mice infected with mutants had lower splenic weight (Fig. 6B) which corresponded to reduced gross pathology in all spleens infected with ΔBPSS0899 or ΔBPSL1494 and in half of ΔBPSS1321-infected spleens (Fig. 6C). Interestingly, this finding did not correlate with the difference in splenic bacterial burden. Additionally, the lung and liver bacterial burden were not significantly altered by toxin loss (Fig. 6D–F).

**Figure 7:**
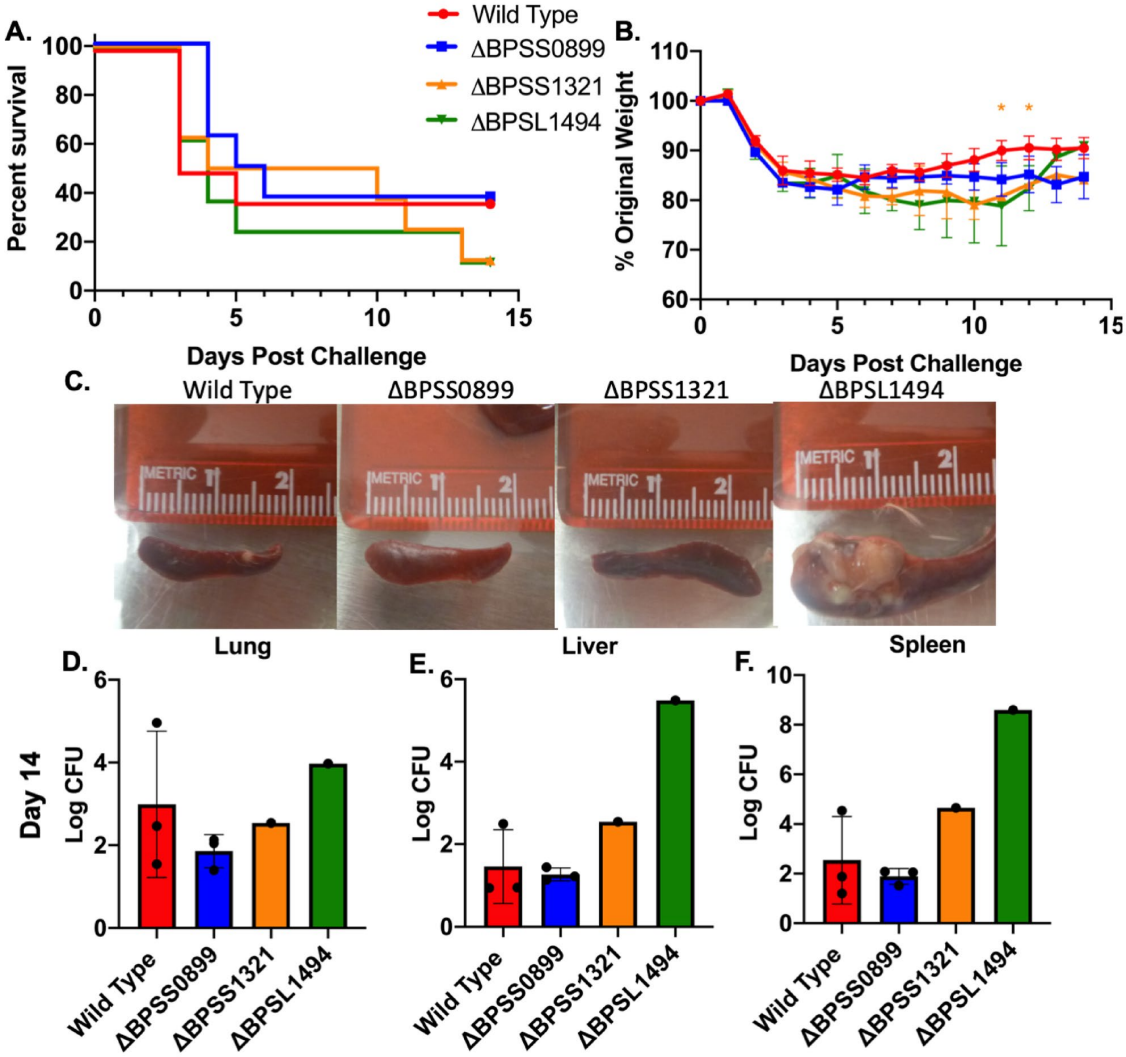
In vivo Persistence of ΔBPSS0899, ΔBPSS1321, and ΔBPSL1494 in the Absence of Treatment. Mice were intranasally infected with 1.5 LD_50_ of wild type or CFU equivalent of each mutant and (**A**) survival and (**B**) weight was monitored for 14 days after which organs of surviving mice were collected, (**C**) visualized for gross pathology, and CFU burden enumerated. (**D**) Lungs, (**E**) livers, and (**F**) spleens were homogenized and bacteria cultured on agar to assess bacterial burden. Statistics were completed using Two-Way ANOVA or One-Way ANOVA. *P < 0.05 and **P < 0.01.

In our in vitro macrophage assays, mutants showed reduced intracellular survival in the absence of antibiotics (Fig. 4B), but not when antibiotics were added (Fig. 4B). To test if colonization was altered in the absence of antibiotics, mice were infected with a low dose (1.5 LD_50_) of each bacterial strain. After challenge, all mice became ill, had weight loss, and some succumbed to infection (Fig. 7A,B). There was no significant difference in survival between mice infected with the respective toxin mutants and compared to those infected with the wild type strain (Fig. 7A). In contrast to the study carried out with antibiotics, infection with ΔBPSL1494 led to large splenic abscesses (Fig. 7C). Quantification of bacterial burden showed no significant difference in the spleens, lungs, or livers when comparing mice (n = 3) infected with wild type and ΔBPSS0899 (Fig. 7D–F). Because we had a single survivor (n = 1) infected with ΔBPSS1321 and ΔBPSL1494, we could not make similar conclusions, but noted that ΔBPSS1321 and ΔBPSL1494 had a splenic burden of 2 and 6 logs higher than the average burden observed with the wild type mice, respectively (Fig. 7D–F).

## Discussion

Due to the likelihood that an intracellular bacteria such as *Bpm* might enter a persistence state, targeting the mechanisms associated with this phenotype offers an opportunity to reduce non-antibiotic resistance treatment failures, infection relapses, and potentially prevents the emergence of new antibiotic resistant strains^12,48^. Although targeting TASs is an ideal approach because it is a bacterial specific mechanism, many pathogens harbor numerous TAS and in many cases they have redundancy in their functions^21,49^. It is also unclear how many toxins are elicited by any given condition and therefore, it is difficult to determine how many TAS play a role in persistence during infection in the host. The approach presented here used known data on toxin families and existing *Bpm* gene expression data to identify and down-select putative toxins based on their association with environmental cues. We found that approximately half of the putative toxins were associated with a wide variety of stress conditions, including antibiotic treatment, supporting the idea that multiple TAS might be required and their functions overlapped to achieve a persistent phenotype. This data supports previous findings indicating that free-living organisms have more toxins, likely to promote adaptation to changing surroundings^49^. This observation is also true for *Bpm* because: (a) this pathogen is a soil and water dwelling organism and many of the conditions evaluated are likely found during environment changes and, (b) antibiotics are a strong selective pressure, which are encountered in the environment during interspecies bacterial warfare. In contrast, fewer genes were associated with host-like conditions. These findings are expected because bacteria have been adapting to their environment and competing with other bacteria for a billion years, while bacterial-host interaction has occurred more recently.

The prediction method used here proved to be an efficient method of down-selecting toxins to study. As with any bioinformatic platform there are strengths and weaknesses. Here, min–max normalization was used and resulted in over representation of constitutively active genes. This brought into light a reciprocal correlation between gene expression activity and conservation. It is also important to mention that identification of antitoxins and their association with their respective toxins was not performed in this study because we want to first provide a wide view of the *Bpm* genes sharing features with known toxins. Further, constitutively expressed genes have not been the focus in TAS research. Continuous expression of a gene product is energetically costly to the bacteria and coupled with their high conservation, might suggest that the protein product is important for metabolic/survival purposes. In terms of TAS, being constitutively expressed has the potential to have a protein that can be activated faster and which might induce persistence more readily, therefore the effect of these gene products was the focus of the rest of our study.

Highly conserved, constitutively expressed, toxins BPSS0899, BPSS1321, and BPSL1494 were further investigated in *Bpm* using both over-expression and unmarked mutant strains. All three toxin mutants were defective in responding to one or more host stress stimuli (nutrient deprivation or NO) and had a reduced ability to survive in macrophages, but the survival defect was lost when macrophage persistence was induced using levofloxacin. These findings indicate that the BPSS0899, BPS1321, and BPSL1494 toxins might played a role in surviving the harsh environment of the host cell, but their absence may be compensated by activation of other toxins when an antibiotic is added to the medium early during the experiment. The selective activation of persistence by host stress alone is not discussed in the persistence field but may explain why people in endemic areas can be found harboring *Bpm* in the form of abscesses without being symptomatic^50–52^. Additionally, it is speculated that asymptomatic persistent infections provide a continuous immune protection from other *Bpm* infections, which has been corroborated by murine vaccine studies showing that the strongest protection was achieved with the vaccine strains that asymptomatically persists in the host^53,54^. To further examine the role of host-mediated persistence, there is a need for the development of novel methods to examine persistence in the absence of antibiotics.

When persistence was tested in an in vivo murine model of inhalational melioidosis, there was no evident effect on colonization with or without levofloxacin treatment. A surprising observation was that after treatment ΔBPSS0899 and ΔBPS1321 resulted in a reduced gross pathology compared to wild type strain. Conversely, without levofloxacin treatment ΔBPSL1494 had larger splenic abscesses. In the absence of CFU changes, we conclude that the change in gross pathology might be immune-mediated. For a bacterial pathogen such as *Bpm*, whose preferred niche in the host is the macrophage, persistence not only relies on the bacterial but also the host cell states. Previous work has shown that *Salmonella* persisters can modulate macrophage polarization toward a M2 phenotype^55^. For *Bpm*, there are no studies investigating the role of persistence on immune state modulation. Epidemiological and clinical data show that immune dysregulation seen in diabetes is the largest risk factor associated with melioidosis recurrent infections^2^. Future direction should aim to further understand the role of *Bpm* persistence in macrophages, the effect on cellular immune state, and whether *bona fide* TAS are playing a specific role within this environment. Until then, the findings here are intended to shift the paradigm of toxin–antitoxin research by using a data-driven approach to facilitate their identification and supports the utility of big data analysis to investigate constitutively expressed toxins as possible regulators of the persistence phenotype.

## Materials and methods

### Ethics statement

All manipulations with *Bpm* were conducted in CDC/USDA-approved and registered biosafety level 3 (BSL3) facilities at UTMB. Experiments with select agents were performed in accordance with BSL3 standard operating practices. The animal studies were carried out humanely in strict accordance with the recommendations in the Guide for the Care and Use of Laboratory Animals by the National Institutes of Health. The protocol (IACUC #0503014D) was approved by the Institutional Animal Care and Use Committee of the University of Texas Medical Branch.

### Bacterial strains and growth conditions

The bacterial strains used in this study are listed in Supplemental Table S4. *B. pseudomallei* strain K96243 was obtained from BEI Resources, Manassas, VA, USA. *E. coli* and *Burkholderia* strains were stored in LB with 5% glycerol (LBG) at − 80 °C and prior to use strains were streaked onto Luria–Bertani (LB) agar and grown for 36–48 h at 37 °C. For liquid cultures, 3–5 colonies were inoculated into LB broth or RMPI with HEPES and L-glutamine and grown for 16 h at 37 °C with agitation (200 rpm). LBG agar was used in the process of generating isogenic mutants.

### Toxin identification

All known toxins used to identify TAS in *M. tuberculosis* were considered in our identification^21^ (presented in Supplemental Table S1). All bacterial and archaeal sequences were downloaded from the EMBL-EBI Pfam database (https://pfam.xfam.org) for each toxin family. Sequences were PSI-Blast against *Bpm* K96243 (taxid:272560) with an expect cut-off 0.002. Expect value is a parameter that describes the number of hits one can “expect” to see by chance when searching a database, like a p-value. The results were sorted to remove redundancies, expect value further reduced to 0.001, and only toxins below 500 amino acid included^28^. Identified toxins were compared to toxins identified were compared to exiting results from:

RATSA (https://hdl.handle.net/10871/9303),

TASMania (https://shiny.bioinformatics.unibe.ch/apps/tasmania/),

TADB for chromosome one and two (https://bioinfo-mml.sjtu.edu.cn/TADB2/browse_org_result.php?type=genome&org_id=NC_006351) and (https://bioinfo-mml.sjtu.edu.cn/TADB2/browse_org_result.php?type=genome&org_id=NC_006350).

### Toxin conservation

Presence or absence of toxin identified in cluster 1–3 was done using the ortholog data available on the *Burkholderia* Genome Database (during June 2018) (https://burkholderia.com). The analysis is carried out using DIAMOND high throughput protein alignment via translated DNA. For the analysis, the ortholog data for putative toxins was downloaded, sorted by species, and the percent of total strains on the databased used to calculated conservation across *Bpm* (n = 677)*, B. mallei* (65)*, B. thailandensis* (28)*,* and *B. cenocepacia* (243) strains. All genomes were included regardless of their completion. Genomes with homologous genes with an expect value of 0.0001 were considered positives orthologs.

### Repurposing existing data

Gene expression of *Bpm* following exposure to 82 independent conditions was obtained from a previous publication^26^. Median log_10_ raw reads of putative toxins and selected control genes were isolated from the data. The values were normalized on the expression of bacteria in LB at stationary phase (LBS) and min–max normalized presented as a heat map (https://www1.heatmapper.ca/expression/) with Manhattan distance metric clustering.

### Over-expression assays

Selected toxin genes of interest were amplified with primers specified in Supplemental Table S5, cleaned with Qiagen PCR purification kit (Qiagen, Hilden, Germany) then ligated into either pBad-myc-his (Thermo Fisher Scientific, Massachusetts, USA) or pSCRba2 (Addgene.org), using Gibson kit (NEB, Massachusetts, USA). All constructs were transformed into *E. coli* DH5α competent cells (NEB, Massachusetts, USA). Constructs were confirmed with PCR and Sanger sequencing (UTMB Sequencing core). pBAD-myc-his constructs were transferred into *E. coli* DH10B cells which have a disrupted arabinose metabolism pathway (*araD139*; NEB, Massachusetts, USA). pSCRba2 constructs were transferred into *B. thailandensis* E264*.* Bacteria cultures grown in M9, supplemented with 2% glycerol and 1% casamino acids (BD), were diluted and then grown at 37 °C with agitation (200 rpm) until they reached an OD_600_ of 0.2. *E. coli* were induced with 0.2% arabinose or not stimulated, and growth monitored by optical density over time. *B. thailandensis* induced with 0.2% rhamnose or not stimulated and growth monitored at 37 °C with agitation (200 rpm). All experiments were carried out with antibiotics to maintain the plasmids.

Persister assays with *B. thailandensis* E264 were carried out on bacteria collected after 2 h of induction. Bacteria were normalized to the same concentration, exposed to either 5 × or 10 × MIC (MIC = 1 μg) of levofloxacin for 24 h, then quantified and normalized to the input concentration. Data is represented as a fold-change in persistence compared to non-induced bacteria. One-Way-ANOVA statistical analysis was done to examine significant difference between bacteria with empty vector and those expressing a toxin.

### Mutagenesis scheme

The *Bpm* ΔBPSS0899*,* ΔBPSS1321*,* and ΔBPSL1494 mutants were constructed using a parental mating approach using pMo130 as previously described^18,56^. Two fragments were amplified (1) 400–600 base pair upstream and (2) the first 21 base pairs of the toxin gene, and the last 9 base pairs of the toxin gene accompanied by 400–600 bases pairs downstream (primers listed in Supplemental Table S5). The fragments were inserted into pMo130 using Gibson Assembly (NEB, Massachusetts, USA) and transformed into *E. coli* S17-1 λ*pir* and introduce to *Bpm* K96243 via biparental mating. Merodiploids were selected using LBG agar with kanamycin and then plasmid loss was promoted by counter selecting on YT agar supplemented with 15% sucrose to obtain single deletion mutants. The mutations were then confirmed via PCR and Sanger sequencing (UTMB Sequencing core).

### Growth curves

Isolates were grown overnight in LB or RPMI with HEPES (Gibco, Thermo Fisher Scientific, Massachusetts, USA) and the following day, diluted to an OD_600_ of 0.1. Cultures were incubated at 37 °C with agitation (200 rpm) and optical density read over 48 h.

### Swarming assays

Bacterial cultures were diluted 1:10 and 2 μl was spotted in the center of semisolid nutrient agar plate (5 g/l bacto agar, 8 g/l nutrient broth N°2, +/− 0.5% [w/v] glucose). Plates were incubated for 24 h and the diameter of bacterial growth measured and presented as a percentage of the plates’ total diameter which was analyzed with One-Way ANOVA with Kruskal–Wallis correction.

### Traditional persistence assays

Bacterial cultures were adjusted to 1 × 10^8^ CFU/ml in medium containing 100 × MIC of levofloxacin. Cultures were incubated for 24 h at 37 °C without shaking, after which the surviving bacteria were quantified by serial dilution and plating on LB agar. Surviving bacteria were normalizing to the input and analyzed using One-Way ANOVA with Kruskal–Wallis correction.

### Macrophage survival assays

Human U937 pleural monocytic cells (CRL-1593.2) were maintained in Modified RPMI-1640 Medium (ATCC 30–2001, Virginia, USA) supplemented with 10% FBS (Gibco), 1 mM sodium pyruvate (Invitrogen, California, USA), and 1 mM penicillin–streptomycin (Invitrogen). For infection assays, 2 × 10^5^ cells/well were seeded in 24-well cell culture plates and supplemented with 25 ng/ml phorbol myristate acetate (PMA; Sigma, Missouri, USA) to induce macrophage differentiation 72 h prior to infection. Twenty-four h prior to infection, U937 cells were washed once and media replenished without PMA. For M1 differentiation, 16 h prior to infection media was replaced with 10 ng/ml of human IFNγ (Peprotech) supplemented medium. Cells were infected with bacteria at a MOI of 10 and synchronized by centrifugation. After a 30 min invasion, cells were washed with PBS and fresh medium containing 100 μg/ml kanamycin (extracellular restricted) or 80 μg/ml of levofloxacin (20 × MIC; cell permeable). At 24 h post infection, cells were washed with PBS and lysed with 0.1% Triton-X100 (Sigma), serial diluted in PBS, and plated on LB agar. After incubation, colonies were counted, normalized for invasion, and analyzed with One-Way ANOVA with Kruskal–Wallis correction.

### Pre-induced persistence assays

Bacteria were grown in RPMI then adjusted to 5 × 10^7^ CFU/ml in RPMI with 100 µM of spermine NONOate as a NO donor (sNO, Sigma Missouri, USA). Alternatively, bacteria were adjusted to approximately 5 × 10^7^ CFU/ml in minimal medium supplemented with 2% glycerol and 1% casamino acids (M9; Becton Dickinson, New Jersey, USA). After 30 min of exposure at 37 °C without shaking, the samples were diluted 1:1 with medium containing levofloxacin to yield a final concentration of 20 × MIC (80 µg/ml) and in the case of the NO study, additional spermine NONOate to maintain their respective concentration. Bacteria were then incubated for 2 h at 37 °C without shaking and then survival enumerated. Percent survival was determined by normalizing the input after the initial 30 min incubation. Statistical significance was determined using One-Way ANOVA with Kruskal–Wallis correction.

### In vivo bacterial infection model

Female, 6 to 8-week-old BALB/c mice were obtained from Jackson Laboratories (Bar Harbor, ME, USA). Mice were housed in microisolator cages under pathogen-free conditions and provided with rodent feed and water ad libitum and maintained on a 12 h light cycle. Before experiments, mice were given 5 days of acclimation. Anesthetized BALB/c mice (n = 5 per group) were inoculated intranasally (I.N.) with equivalent to 3.5 LD_50_ *Bpm* K96243 wild type (1 LD_50_ = 312 CFU), and CFU equivalent of ΔBPSS0899*,* ΔBPSS1321*,* or ΔBPSL1494 diluted with PBS in a total volume of 50 µL (25 µL/ nare). Mice received daily intraperitoneal injections of levofloxacin (25 mg/kg/day in PBS) starting at 24 h post-infection and continuing for five days. Mice were monitored and weighed daily throughout the 20-day study. For the low-dose infection BALB/c mice were anesthetized (n = 5 per group) and then inoculated intranasally (I.N.) with equivalent to 1.5 LD_50_ *Bpm* K96243 wild type, ΔBPSS0899*,* ΔBPSS1321*,* or ΔBPSL1494 and monitored and weighed daily throughout the 14-day study. Humane endpoints were strictly observed, and time of death was recorded upon animals succumbing to infection or at the study’s endpoint. Survival curves were analyzed by using the Kaplan–Meier method with log-rank test. For CFU enumeration, animals were euthanized, their lungs, liver, and spleen collected, and homogenized using Covidien Precision tissue grinders (Thermo Fisher Scientific, Massachusetts, USA). Tissue homogenates were serially diluted in PBS, plated, and incubated for 48–72 h at 37 °C. Colonies were counted and normalized to organ weight (g) and significance determined using T-test with a Mann–Whitney correction or One-way ANOVA depending on the number of groups.

## Supplementary information

Supplementary information.
