# Supplementary material for: Predicting toxins found in toxin–antitoxin systems with a role in host-induced Burkholderia pseudomallei persistence
Source: Sci Rep. 2020 Oct 9;10:16923. doi: 10.1038/s41598-020-73887-3 (PMC7547725; doi:10.1038/s41598-020-73887-3)
Supplement: Supplementary file 1 — Supplementary information. [file 41598_2020_73887_MOESM1_ESM.docx]

**Supplemental Data**

Predicting Toxins found in Toxin-Antitoxin Systems with a Role in Host-Induced *Burkholderia pseudomallei* Persistence

Brittany N. Ross ^a†^, Joseph D. Thiriot ^a^, Shane M. Wilson ^a^, Alfredo G. Torres ^a,b^**^*^**

^a^ Department of Microbiology and Immunology, ^b^ Department of Pathology, University of Texas Medical Branch, Galveston, Texas, 77555

**^*^** Corresponding author:

Phone 409-747-0189

Fax 409 747 6869

altorres@utmb.edu

^†^ Present address: Center for Microbial Dynamics & Infection, School of Biological Sciences, Georgia Institute of Technology, Atlanta, GA 30332, USA

**Supplemental Table S1. Type II Toxin-Antitoxin Families**


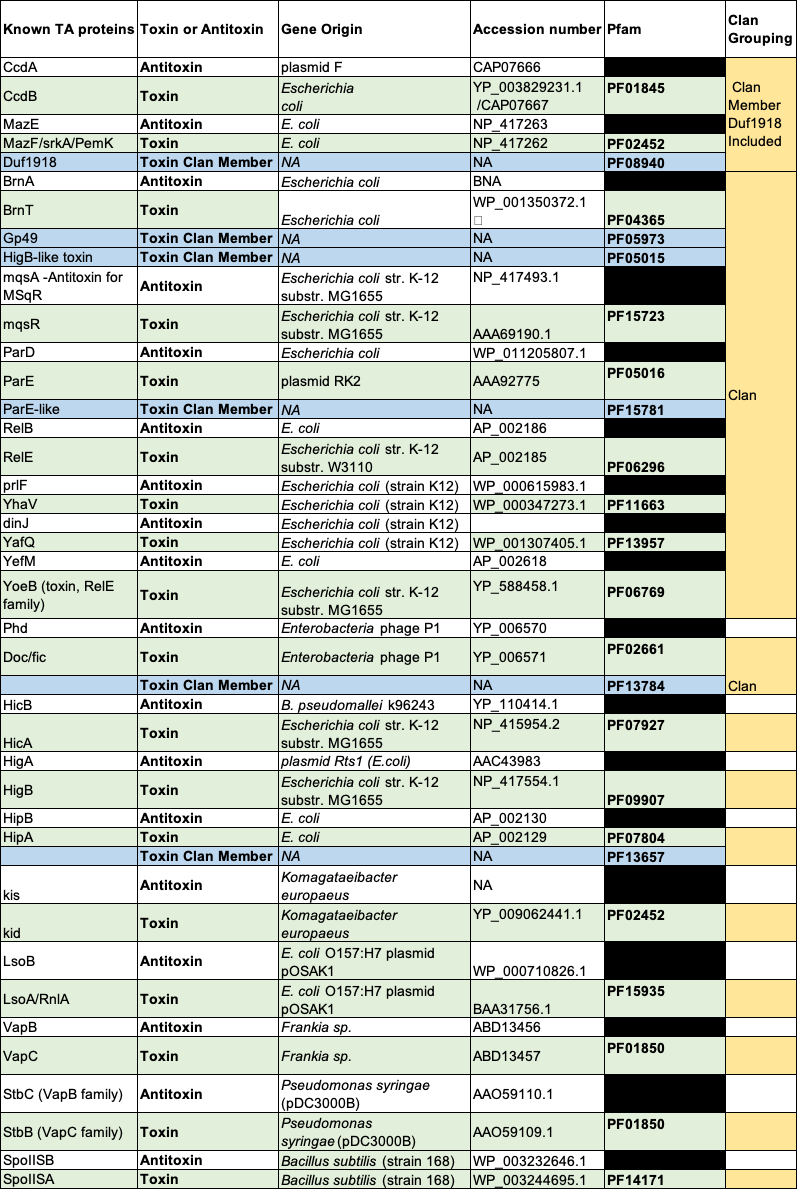


Adapted from Ramage *et al.,* 2009. Footnote: Pfams are grouped by clans. If a clan included other Pfams that represented toxins or portions of toxins (N- or C- terminus) they were included, and gene origin and accession number were designated as not applicable (NA).

**
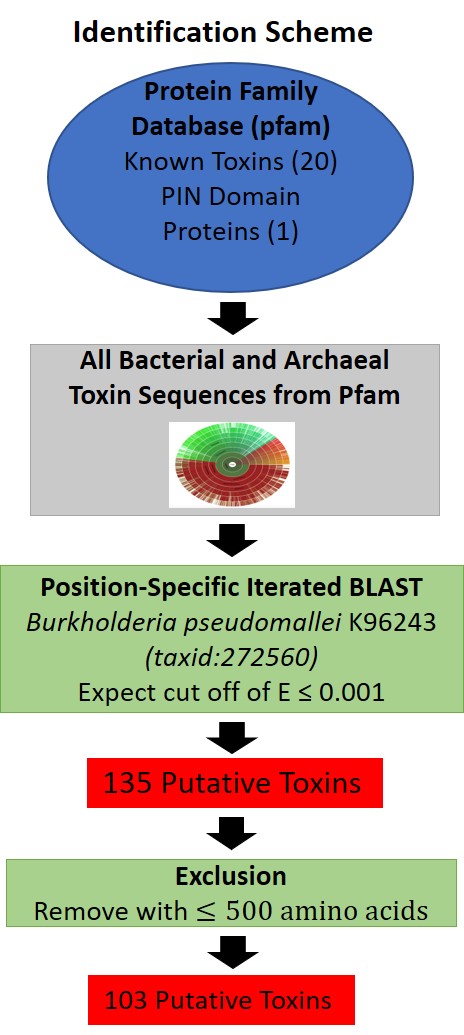
**

**Supplemental Figure S1. Identification Scheme of Putative *Bpm* Toxins.**

Toxins were identified by taking the pfam file containing all known toxins of a given family and blasting against Bpm K96243. Identified genes were excluded if they had an expect value above 0.001 and were greater than 500 amino acids.


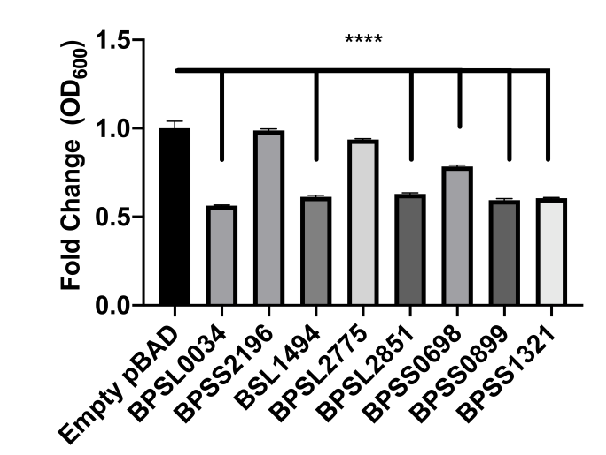


**Supplemental Figure S2.** **Identified Toxin Functionality.**

E. coli DH10B carrying empty pBAD plasmid or pBAD carrying a putative toxin gene were grown to an OD_600_ of 0.2 and induced with 0.2% arabinose. After 7 h, the OD_600_ was taken and normalized to E. coli carrying the empty plasmid. ****p<0.0001.

**Supplemental Figure S3. Overlap of Identified Toxins with Other Platforms.**

The number of toxins found in this study was compared to putative toxins found by both TASmania and RASTA. The data is presented based on chromosomal location, (A) chromosome 1 and (B) chromosome 2.

**Supplemental Table S2. Growth conditions affecting *Bpm* and its mutants.**


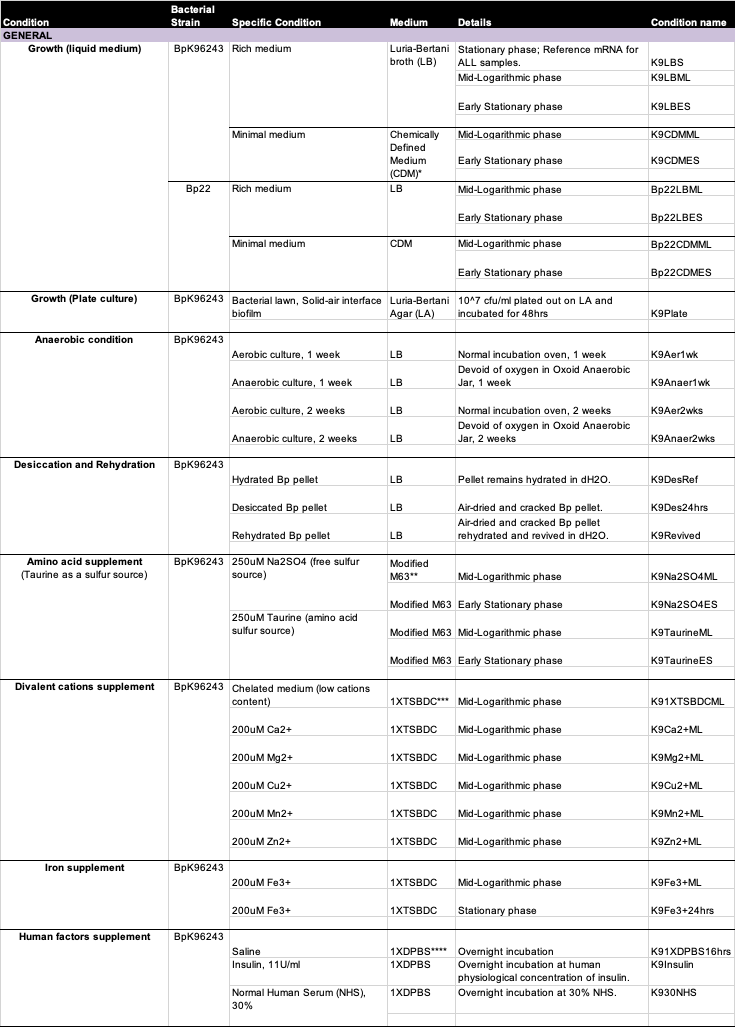


**Supplemental Table S2. Growth conditions affecting *Bpm* and its mutants Continued**
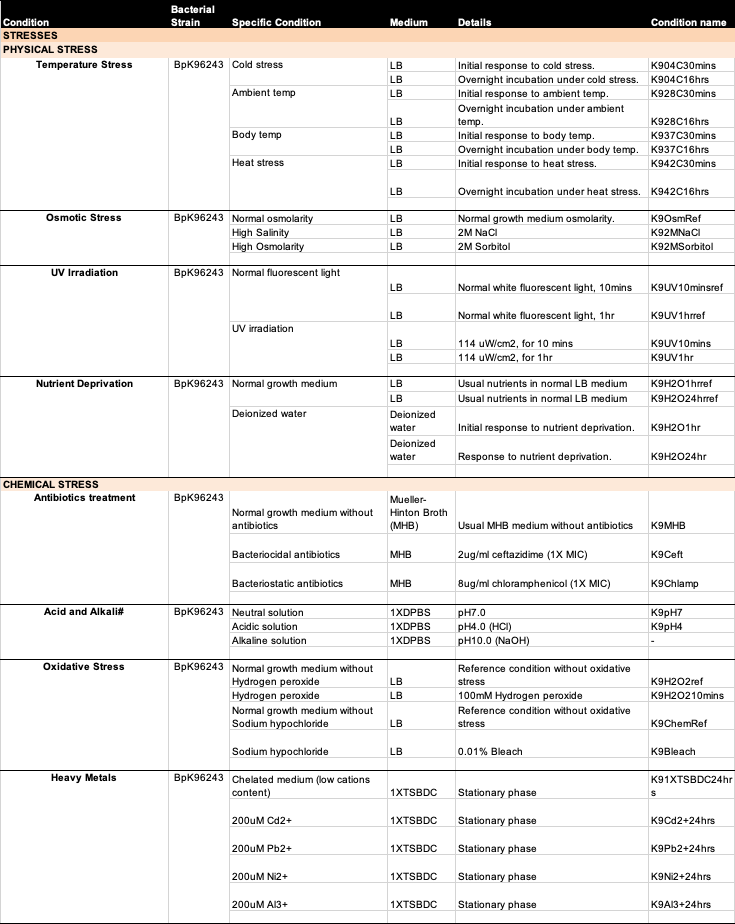
 **Supplemental Table S2. Growth conditions affecting *Bpm* and its mutants Continued**
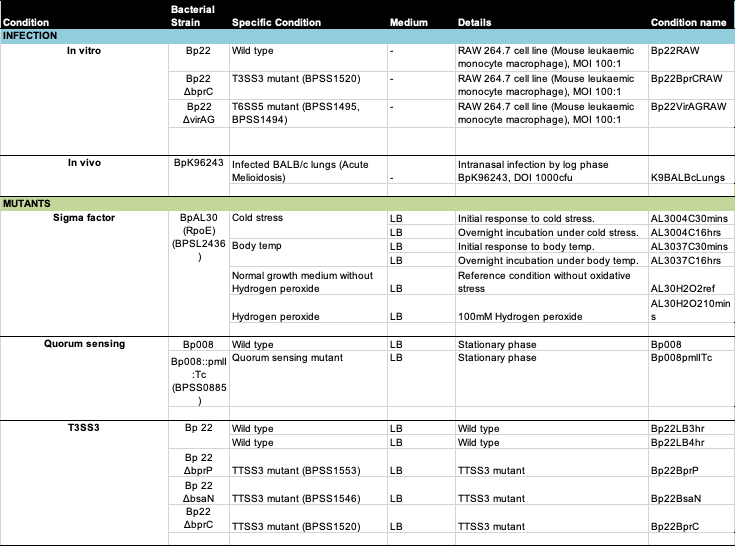


Footnote: This Table was adapted from Ooi *et al.,* 2011.

**Supplemental Table S3. Putative Toxins Function and Operon.**


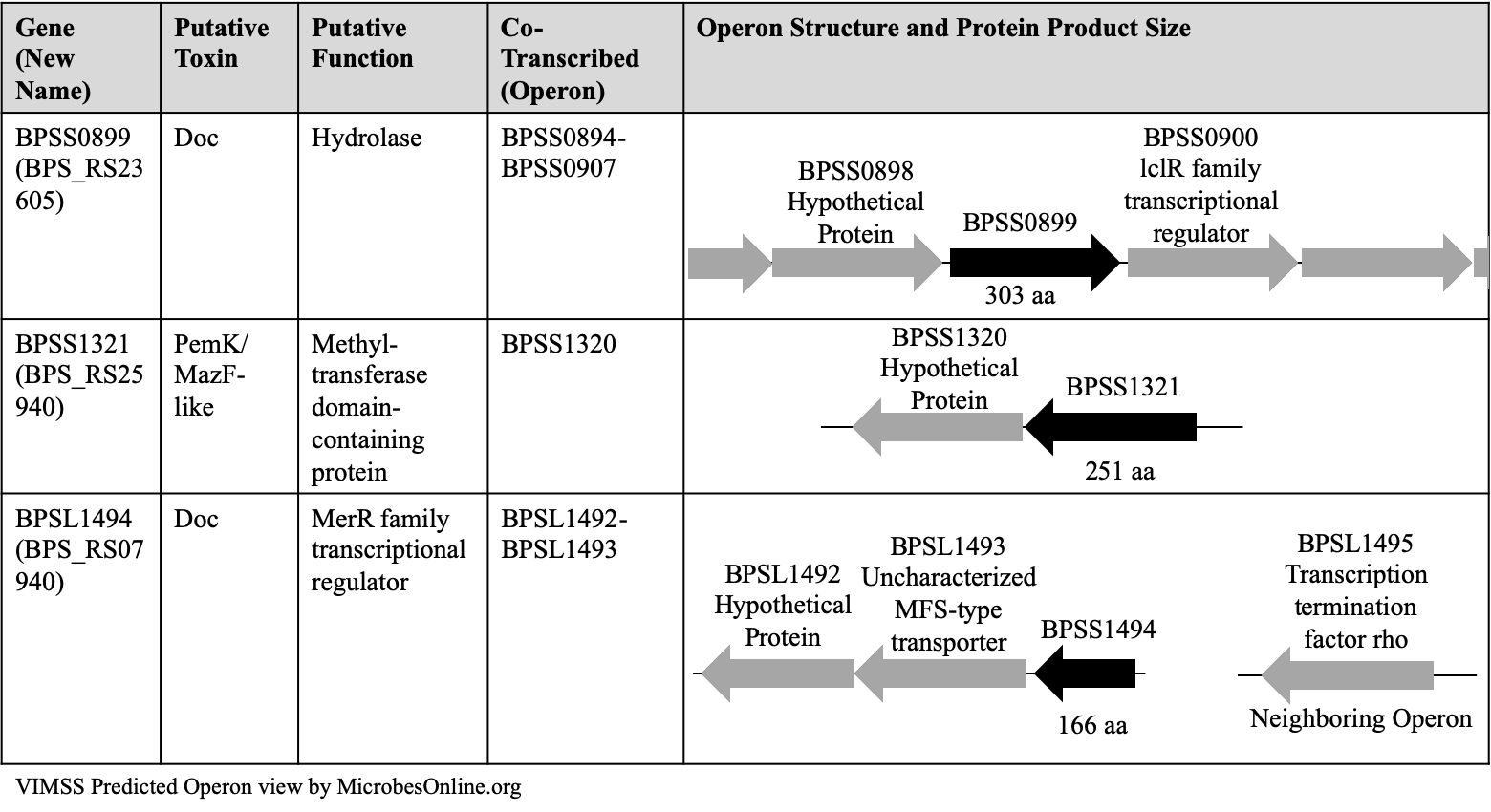


***
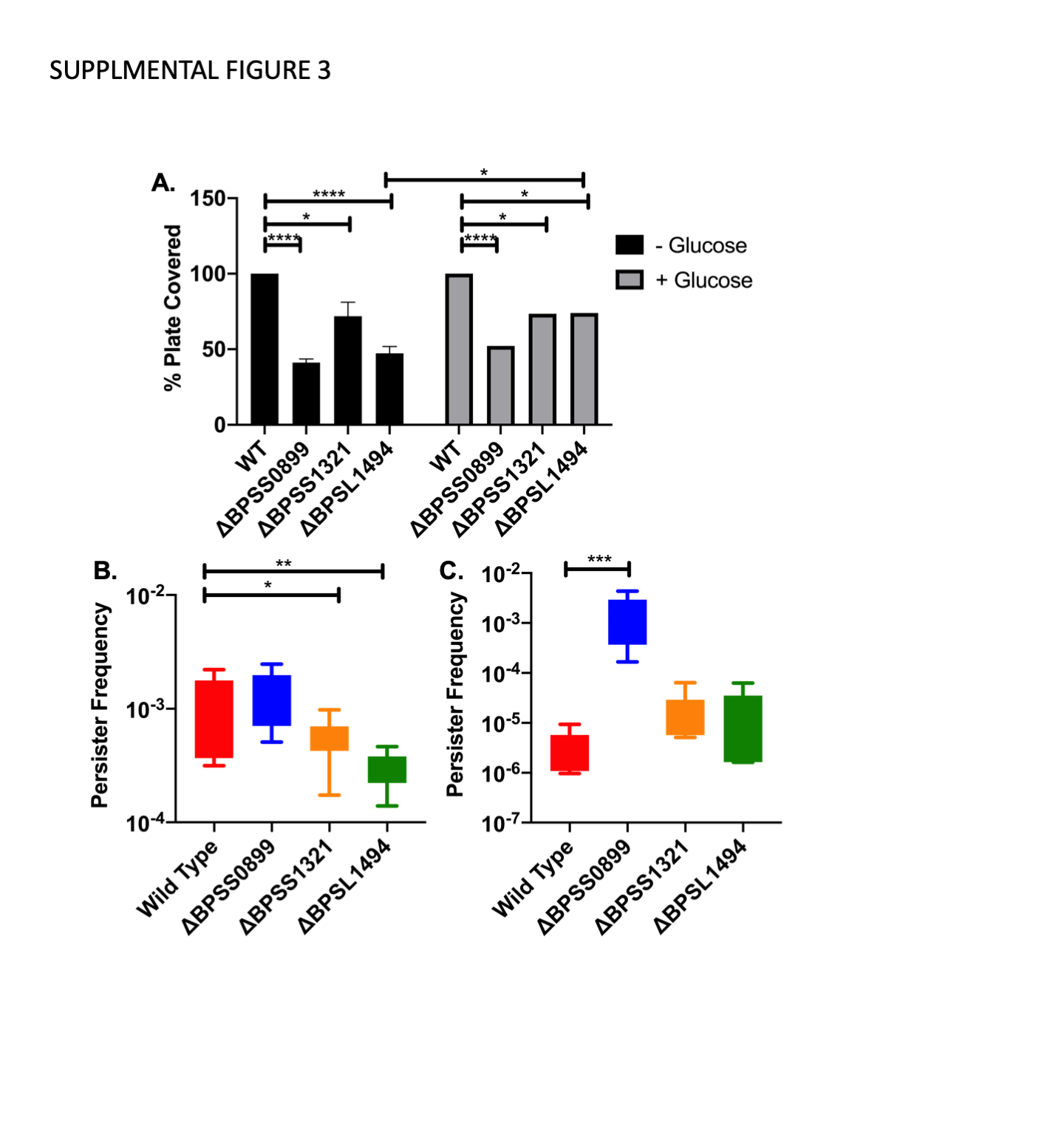
***

**Supplemental Figure S4*.* Phenotypic Characterization of Toxin Mutants**

Reduced swarming motility is linked to persistence. (A) To test swarming, bacteria were spotted on the center of a semi-solid agar plate with or without glucose supplementation and diameter of swarming measured after 24 h of incubation at 37*°*C. Antibiotic induced persistence assays were carried out on Bpm K96243 wild type and toxin mutants ΔBPSS0899, ΔBPSS1321 and ΔBPSL1494 grown in either (B) LB or (C) RPMI, and then exposed to 20x MIC of levofloxacin for 24 h. The persistence frequency represents the ratio of bacteria surviving the antibiotic treatment compared to the input. * P<0.05, ** P<0.01, *** P<0.001, **** P<0.0001.

**Supplemental Table S4. Bacterial Strains**

| **Strains** | |
| --- | --- |
| *B. pseudomallei* K96243 | BEI Resources (Manassas, VA, USA) |
| *B. pseudomallei* K96243 ∆BPSS0899 | This publication |
| *B. pseudomallei* K96243 ∆BPSS1321 | This publication |
| *B. pseudomallei* K96243 ∆BPSL1494 | This publication |
| *E. coli* S17 λ*pir* pMO130∆0899 | This publication |
| *E. coli* S17 λ*pir* pMO130∆1321 | This publication |
| *E. coli* S17 λ*pir* pMO130∆1494 | This publication |
| *E. coli* DH10B pBAD Empty | This publication |
| *E. coli* DH10B pBAD BPSS0899 | This publication |
| *E. coli* DH10B pBAD BPSS1321 | This publication |
| *E. coli* DH10B pBAD BPSS2196 | This publication |
| *E. coli* DH10B pBAD BPSL2775 | This publication |
| *E. coli* DH10B pBAD BPSL1494 | This publication |
| *B. thailandensis* E264 | American Type Culture Collection (ATCC) |
| *B. thailandensis* pScrhaB2 Empty | This publication |
| *B. thailandensis* pScrhaB2 BPSS0899 | This publication |
| *B. thailandensis* pScrhaB2 BPSS1321 | This publication |
| *B. thailandensis* pScrhaB2 BPSL1494 | This publication |

**Supplemental Table S5: Primers used in this Study**

| **Primers** |  |
| --- | --- |
| pBAD BPSS0899 Sac1 F | aaagagctcgatgagcacgatttccac |
| pBAD BPSS0899 Knp1 R | tttggtacctcatggtgtgcctcgcga |
| pBAD BPSS2775 Sac1 F | aaagagctcgatgcgcacgacgctgaaacgg |
| pBAD BPSS2775 Knp1 R | tttggtacctcatgctgcgctctgctcctt |
| pBAD BPSL1494 Sac1 F | aaagagctcgatgagcgatgccccgcccacc |
| pBAD BPSL1494 Knp1 R | tttggtacctcatgcgcttgtcccgcccgt |
| pBAD BPSS2196 Knp1 F | aaaggtaccatgtcattgcgagaccagatg |
| pBAD BPSS2196 HindIII R | tttaagcttttacgccacttcgaagcggcg |
| BPSS0899 pSCrhaB2 F | tgaaattcagcaggatcacatatgagcacgatttccacgacg |
| BPSS0899 pSCrhaB2 R | ctcatccgccaaaacagccaagctcatggtgtgcctcgcgatc |
| BPSS1321 pSCrhaB2 F | tgaaattcagcaggatcacatatgttgcgcatcgacagaatc |
| BPSS1321 pSCrhaB2 R | ctcatccgccaaaacagccaagctcaagcgctccccagaatc |
| BPSL1494 pSCrhaB2 F | tgaaattcagcaggatcacatatgagcgatgccccgccc |
| BPSL1494 pSCrhaB2 R | ctcatccgccaaaacagccaagctcatgcgcttgtcccgcc |
| pMO130∆BPSS0899 1F | gagctgatatcagggccccgctagcgggcgaaagcttcgccca |
| pMO130∆BPSS0899 1R | ctcatggtgtcgtcgtggaaatcgtgctcatc |
| pMO130∆BPSS0899 2F | ttccacgacgacaccatgagcgagccgc |
| pMO130∆BPSS0899 2R | cagctcaagcttcccgggaagatctgcgttttgcgccttcgcg |
| pMO130∆BPSS1321 1F | gagctgatatcagggccccgctagcgcgaaatgggcgtccggc |
| pMO130∆BPSS1321 1R | ctcaagcgctgattctgtcgatgcgcaatcgc |
| pMO130∆BPSS1321 2F | cgacagaatcagcgcttgaggagatgttc |
| pMO130∆BPSS1321 2R | gattaattgtcaacagctcaagcttgtacagatcgggaatcacc |
| pMO130∆BPSL1494 1F | gagctgatatcagggccccgctagccttcggcgcggcgcgcaa |
| pMO130∆BPSL1494 1R | ctcatgcgctggtgggcggggcatcgctc |
| pMO130∆BPSL1494 2F | cccgcccaccagcgcatgagtgccgcgc |
| pMO130∆BPSL1494 2R | gattaattgtcaacagctcaagcttgcgcccatcatggggccg |
